# Complete genome sequence of the *Sphingobacterium bambusae* type strain KACC 22910^T^

**DOI:** 10.1128/mra.00871-24

**Published:** 2024-10-22

**Authors:** Do-Hyun Kim, Hyorim Choi, Jun Heo

**Affiliations:** 1 Agricultural Microbiology Division, National Institute of Agricultural Sciences, Rural Development Administration, Wanju-gun, Jeollabuk-do, South Korea; 2 Division of Biotechnology, Jeonbuk National University, Iksan-si, Jeollabuk-do, South Korea; University of Strathclyde, Glasgow, United Kingdom

**Keywords:** type strain, KACC, *Sphingobacterium bambusae*

## Abstract

We report the whole genome sequence of *Sphingobacterium bambusae* KACC 22910^T^. The complete genome consists of a 5.6 Mb circular chromosome with a G + C content of 44.4 % and 4,526 predicted coding genes.

## ANNOUNCEMENT

The genus *Sphingobacterium* was first described by Yabuuchi et al. (1) and currently comprises 71 validly published species (https://lpsn.dsmz.de/genus/sphingobacterium). Many of these species have been isolated from soil environments. Among them, *S. bambusae* was initially isolated from a bamboo plantation by (2). However, no genomic data for this species have been registered. To address the lack of genomic data, we obtained KACC 22,910*^T^*, which is a type strain of *S. bambusae,* and performed a complete genome analysis to establish a foundational data set for functional genomics research on soil biodiversity.

Here, we report the whole genome sequence of *S. bambusae* KACC 22910^T^. The strain was obtained from the Korean Agricultural Culture Collection (KACC). It was cultured on Nutrient Agar (NA) medium (BD Difco, NJ, USA) with pH 6.0 at 28℃ for 4 days. According to the manufacturer’s protocol, the Qiagen MagAttract HMW DNA kit (Qiagen, Hilden, Germany) was used for genomic DNA extraction, and the same DNA products were used for genome sequence analysis on an Illumina NovaSeq 6000 (Illumina, CA, USA) and PacBio Sequel IIe (Pacific Biosciences, CA, USA), respectively. Genomic DNA libraries were prepared using the TruSeq Nano High Throughput Library Prep Kit (Illumina, CA, USA). Libraries of 7–12 kb templates were generated for sequencing with the PacBio SMRTbell prep kit 3.0. Subsequently, they were analyzed using the Sequel II Bind Kit 3.2 and Int Ctrl 3.2. Sequencing was carried out using Sequel II Sequencing Kit 2.0 and SMRT cell 8M trays. HIFI reads were obtained from the PacBio Sequel IIe system and assembled using the microbial assembly application in Flye v2.8.3 (3), based on the Hierarchical Genome Assembly Process (HGAP) (4). The Illumina raw reads of which 90% of the bases had a phred score of 30 or higher were filtered, and adapter trimming was performed using Trimmomatic 0.38 (5). Then, the assembly that was constructed using long-read data was corrected three times using the trimmed Illumina reads through Pilon v1.21 (6). Only one contig was generated as a result of the assembly with the Flye and successfully circularized with the Circlator v1.5.5 (7). Gene prediction and annotation were conducted by the NCBI Prokaryotic Genome Annotation Pipeline v6.4 (PGAP) (8). All tools were executed with default parameters unless stated otherwise.

The Illumina Novaseq 6000 and PacBio Sequel IIe sequencing generated 12,808,047 read pairs, a total of 25,408,064 reads (2 × 150 bp) and 888,234 long reads (9,015,135,467 bp; 12,352 *N_50_*), respectively. A circular chromosome of *S. bambusae KACC 22,910^T^* was assembled with a coverage of 200× and G + C content of 44.4% without plasmid. The chromosome is predicted to contain 4,526 total coding sequences, 18 rRNA genes, 65 tRNA genes, three non-coding RNA genes, and 11 pseudogenes.

## Data Availability

The complete genome of *Sphingobacterium bambusae* KACC 22910^T^ was deposited in NCBI GenBank under the BioProject accession number PRJNA1036373, the BioSample accession number SAMN38124167, and the GenBank accession number CP138332. The raw Illumina and PacBio reads can access the NCBI’s SRA accession number SRR30105955 and SRR29094106, respectively.
